# Pacemaker Prevention Therapy in Drug-refractory Paroxysmal Atrial Fibrillation: Reliability of Diagnostics and Effectiveness of Prevention Pacing Therapy in Vitatron^™^ Selection^®^ Device

**Published:** 2006-04-01

**Authors:** Paolo Terranova, Paolo Valli, Peppino Terranova, Simonetta Dell’Orto, Enrico Maria Greco

**Affiliations:** 1 Divisione di Cardiologia, Azienda Ospedaliera “Luigi Sacco” – Polo Universitario, Istituto di Scienze Biomediche LITA; University of Milan, Italy; 2 Unità Operativa di Cardiologia, Presidio Ospedaliero “Causa Pia Ospedaliera Uboldo”, Cernusco sul Naviglio, Azienda Ospedaliera di Melegnano, Milano

**Keywords:** Sick Sinus Syndrome, Atrial Fibrillation, Atrial Pacing, Pacing Algorithms, Non-Pharmacological Therapy

## Abstract

**Introduction:**

Atrial fibrillation (AF), the most common and rising disorder of cardiac rhythm, is quite difficult to control and/or to treat. Non pharmacological therapies for AF may involve the use of dedicated pacing algorithms to detect and prevent atrial arrhythmia that could be a trigger for AF onset. Selection 900E/AF2.0 Vitatron DDDRP pacemaker (1) keeps an atrial arrhythmia diary thus providing detailed onset reports of arrhythmias of interest, (2) provides us data about the number of premature atrial contractions (PACs) and (3) plots heart rate in the 5 minutes preceding the detection of an atrial arrhythmia. Moreover, this device applies four dedicated pacing therapies to reduce the incidence of atrial arrhythmia and AF events.

**Aim of the Study:**

To analyze the reliability to record atrial arrhythmias and evaluate effectiveness of its AF preventive pacing therapies.

**Material and Methods:**

We enrolled 15 patients (9 males and 6 females, mean age of 71±5 years, NYHA class I-II), with a DDDRP pacemaker implanted for a “bradycardia-tachycardia” syndrome, with advanced atrioventricular conduction disturbances. We compared the number and duration of AF episodes’ stored in the device with a contemporaneous 24h Holter monitoring. After that, we switched on the atrial arrhythmias detecting algorithms, starting from an atrial rate over 180 beats per minute for at least 6 ventricular cycles, and ending with at least 10 ventricular cycles in sinus rhythm. Thereafter, in order to evaluate the possible reduction in PACs number and in number and duration of AF episodes, we tailored all the four pacing preventive algorithms. Patients were followed for 24±8 months (from 20 to 32 months).

**Results:**

All 59 atrial arrhythmia episodes occurred in the first part of this trial, were correctly recorded by both systems, with a correlation coefficient (r) of 0.96. During the follow-up, we observed a significant reduction not only in PACs number (from 83±12/day to 2.3±0.8/day) but also in AF episodes (from 46±7/day to 0.12±0.03/day) and AF burden (from 93%±6% to 0.3%±0.06%). An increase in atrial pacing percentages (from 3%±0.5% to 97%±3%) was also contemporaneously observed.

**Conclusion:**

In this pacemaker, detection of atrial arrhythmia episodes is highly reliable, thus making available an appropriate monitoring of heart rhythm, mainly suitable in AF asymptomatic patients. Moreover, the significant reduction of atrial arrhythmia episodes indicates that this might represent a suitable therapeutic option for an effective preventive therapy of AF in paced brady-tachy patients.

## Introduction

Atrial fibrillation (AF) represents one of the major clinical, social and economical medical challenge [1,2]. Progressive ageing is associated with an inevitable rising in incidence and prevalence of this rhythm disorder [3,4], which causes not only impairments in functional capability being associated to a greater incidence of cerebro-vascular events, but also a rising request for emergency room visits and hospital admission, mainly in patients affected by left ventricular dysfunction or by other significant cardiovascular diseases.

Different therapeutic strategies, both pharmacological and non pharmacological, are available to restore sinus rhythm, prevent arrhythmia recurrences and cardio-embolic events and control the mean ventricular rate [1,2,5]. What is still a challenge is how to tailor appropriate strategy and therapy for each patient.

It is an affirming concept that AF should not be considered as a simple cardiac rhythm disturbance, but rather as a syndrome with a variety of clinical presentations and two frequently negative hemodynamic feature: (1) the lack of atrial contribution to ventricular filling and (2) the irregularity and/or the fast shortening of cardiac cycle lenght [1,2,5,6]. The abnormal automaticity of pulmonary veins’ foci [7] represents a critical determinant for AF onset. It’s well known, nowadays, that a (1) critical atrial mass is necessary to maintain the re-entry atrial circuits [8] and that (2) the adrenergic activation determines a pro-arrhythmic effect on bursts originating from the pulmonary veins [9] or on arrhythmia recurrence after a cardioversion [10]. Notwithstanding, it’s almost impossible to figure out a temporal prevision of the evolution of this “AF syndrome” in each patient.

In 1995, Wijffels and coll. [11] showed, for the first time, that AF causes several changes in atrial electrophysiological properties that could be responsible for the clinical progression and maintenance of this tachyarrhythmia. The persistence of electrical disorders was found to be proportional to the duration of atrial high-rate pacing [8-12].

There are three main electrophysiological assumptions [12-24] for pacing prevention of AF widely accepted:

1. to reduce the arrhythmogenetic effects of bradycardia and, in particular, the atrial refractory periods dispersion;
2. to override atrial premature contractions (PACs), as a possible cause of AF onset;
3. to reduce the compensatory pauses after an atrial or a ventricular premature contraction, thus reducing the dispersion of atrial refractoriness induced by the “short cycle – long cycle” mechanism.

A prospective randomised study from Andersen and coll. [15] on 225 patients with sick sinus syndrome, showed that atrial pacing (AAI) not only significantly reduced the incidence and burden of AF and thromboembolisms when compared with ventricular one, but also that there was a significant reduction in total and cardiovascular mortality.

To improve the capability to prevent the onset of AF, other several approaches have been tested. In patients with marked intraatrial conduction period delays, Saksena [16] and Daubert [17] suggested to resynchronize both atria with dual-site atrial pacing, in different stimulation sites, in order to reduce the consequent atrial electrical abnormalities. Nowadays, this approach has been less considered, because of a low efficacy on AF incidence and burden, with frequent episodes of cross-talk and interference due to the dipole wide range, and because of the higher threshold needed for left atrial pacing. As alternative solutions, Padeletti and coll [18]. suggested the use of a single atrial lead on atrial septum. Several studies are still ongoing, with contrasting preliminary results.

It’s also well known that variability in the origin of PACs should increase the probability of a re-entry mechanism, mainly in presence of an anomalous substrate. On this account, it has been proposed to reduce atrial rate variability using an overdrive atrial steady stimulation alone, although this approach did not obtain significant results. Alternatively, other pacing techniques such as bursts and drives algorithms have been proposed. Several trials [19-24] have been done to evaluate the safety and efficacy of these devices in terminating spontaneous atrial tachyarrhythmias, but the results were quite disomogeneous and so this approach is far from being definitively accepted.

In particular, the Atrial Dynamic Overdrive Pacing Trial (ADOPT) [25] evaluated the effects of atrial overdrive pacing algorithms in 319 patients (mean age 71±10 years) with paroxysmal atrial fibrillation (3-3.5 documented atrial fibrillation episodes in the 12 weeks prior to implant). Atrial pacing reduced symptomatic atrial fibrillation burden (On: 1.9% vs. Off: 2.5%). Symptomatic atrial fibrillation burden decreased over time for both groups. No significant difference was noted in the number of atrial fibrillation episodes (On: 3.2 ± 8.6 vs. Off: 4.3 ± 11.5) or total hospitalizations (On: 9% vs. Off: 13%) between the two groups. Quality-of- life improved in both groups.

Moreover, in the ATTEST study [26] was evaluated the effectiveness of an atrial therapy device utilizing preventative and antitacycardia pacing in patients with symptomatic atrial fibrillation. The implanted device was an AT500 (Medtronic Inc, Minneapolis, MN) DDDR pacemaker, with a mode switching algorithm (DDIR) during atrial tachyarrhythmias. Atrial antitachycardia pacing was available in rate adaptive burst or ramp modes, and the device also could employ 3 preventative atrial pacing algorithms to attempt to prevent AF/AT recurrences. After a one-month run in period, patients were randomized to either atrial prevention or termination on or off, and followed for three months. At the end of the trial, there was observed no significant difference between the two groups in the run in period or in follow up in either frequency, burden or symptomatic frequency of AT/AF. The efficacy of device ATP was 54% in converting atrial arrhythmias to sinus rhythm as evaluated by the device and the accuracy of the detection algorithm was 99.9%.

Based on the hypothesis of the effectiveness of a non-pharmacological antiarrhythmic electrical therapy, our study was aimed at evaluate the reliability of the four specific pacing algorithms of the DDDRP pacemaker, the Selection^®^ 900E/AF2.0 Vitatron^™^, and to reduce the trigger mechanisms, possibly responsible for AF onset.

## Materials and Methods

We initially evaluated the atrial arrhythmia recording reliability in 15 patients, implanted with a Selection™ 900E/AF2.0 Vitatron® device, for a “brady-tachy” syndrome, with advanced atrioventricular conduction disturbances. Patients were enrolled in a period of 10 months, starting from January 2000 and ending in October 2000. Patients were 9 males and 6 females, with a mean age of 71±5 years, without any previous history of myocardial infarction, angina, diabetes and other known risk factors and in I NYHA functional Class. All patients were initially under oral anticoagulant therapy (INR 2.0-3.0) and IC antiarrhythmic drug therapy (Acetate Flecainide 200 mg per day) for at least 6 months prior to implant. Therapies were not discontinued during the follow-up.

In order to evaluate the Selection™ 900E/AF2.0 Vitatron® atrial arrhythmia recording reliability, we compared the number of atrial arrhythmic episodes and their onset and duration,properly stored in the pacemaker, with a contemporaneous 24h standard Holter cassette recording (Ela Medical™ Synetec® System, version 1.20). A statistical correlation analysis was performed. The pacemaker parameters were settled in order to record atrial arrhythmic episodes lasting at least 6 ventricular cycles for their onset and 10 ventricular cycle for their ending. The atrial cut-off detection rate was of 180 atrial waves per minute. Clinical and follow-up features of our population are summarized in Table 1. Data are expressed as mean ± SEM. Paired t-Student test was performed.

The second aim was a middle-term efficacy evaluation (mean follow up 24±8 months, i.e. from 20 to 32 months) of all the four Selection™ 900E/AF2.0 Vitatron® available pacing algorithms, in order to prevent the trigger mechanisms, possibly responsible for AF onset. Each pacing algorithm was tailored according to the clinical and electrophysiological features of each patient, according to the previously recorded atrial arrhythmic events.

The four used atrial pacing algorithms were the following four:

1. ***Pace Conditioning^™^*** this algorithm consist on a permanent overdrive atrial pacing with an atrial pacing rate of about 15 b.p.m. higher than the beneath intrinsic effective atrial rate.
2. ***PAC Suppression^™^*** this algorithm is designed to reduce the incidence of atrial tachyarrhythmia by a temporary stable atrial overdrive pacing following a PAC. This algorithm provides a temporary atrial overdrive pacing lasting for 600 ventricular cycles after a sensed PAC. At the end of this 600 ventricular cycles, the pacing rate progressively reduces till the lower rate limit or till the emergence of a stable sinus rhythm.
3. ***Post-PAC Response^™^*** this algorithm is designed to reduce the post-extrasystolic pauses by controlling the atrial rate in the 2 beats after a PAC. The first atrial paced beat’s rate is determined by an averaging between the previous physiologic RR interval and the PAC pairing rate. From the second beat onwards, the atrial fleeing rate returns to the atrial physiologic rate.
4. ***Post-Exercise Rate Control^™^*** the post-exercise rate control has been specifically designed to prevent a too fast lowering of heart rate after a physical activity. The post-exercise rate increases proportionally to the difference between the physiological heart rate and the target heart rate, i.e. 90% of the physiological heart rate.

## Results

**Figure 1:**
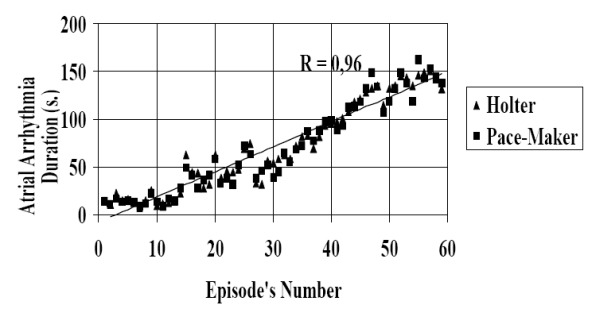
Concordance Between Holter and Pacemaker Recordings

During the first part of this trial, i.e. that comparing Holter recordings and pacemaker storage data, we observed 59 episodes of paroxysmal AF, lasting from some seconds to some minutes, with a highly significant correlation coefficient between Holter and pacemaker recordings (r=0.96) (Figure 1). The AF mean duration was of 70,9±46,8 s. in the Holter group and of 69,6±47,4 in pacemaker recordings (p = 0.881; 95% confidence interval: -18.48 to 15.88). Differences between timing and duration of the two recordings is likely to be due to the cassette tracking speed features.

**Figure 2:**
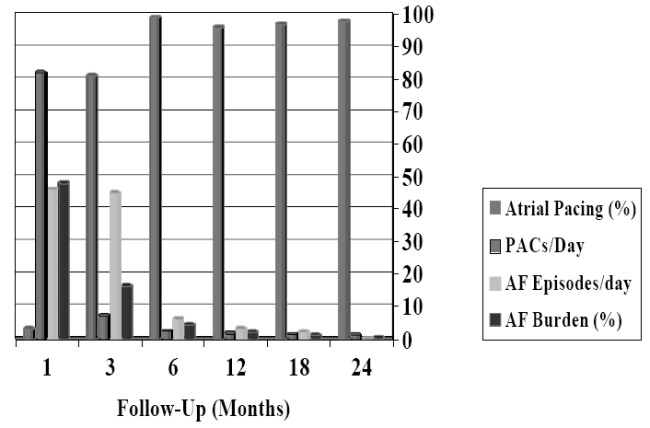
Supraventricular Tachyarrhythmia Reduction After Switching on the 4 Pacing Preventive Therapies

As shown in Figure 2, during the second half of our trial, the antiarrhythmic algorithms produced a significant and progressive reduction in AF episodes (from 46±7 episodes/day to 0,12±0,03 episodes/day; p<0.001; 95% confidence interval: 41.69 to 50.07) and in AF burden (from 93%±6% to 0,3%±0,06%; p<0.001; 95% confidence interval: 89.53 to 95.87), with a concomitant increase in atrial pacing percentage (from 3%±0.5% to 97%±3%; p<0.001; 95% confidence interval: -95.61 to -92.39). AF burden was evaluated and measured during the follow up period using the pacemaker storage data, and no comparison were made with AF burden prior to implant, because of the lack of a real value of this data in our patients, that were sometimes asymptomatic ones. We observed also a decrease in premature atrial contractions from 83±12 PACs/day to 2,3±0,8 (p<0.001; 95% confidence interval: -87.06 to -74.34) (Figure 2). The same method was used to record APCs in the pre- and post- implant period, i.e. the Holter monitoring, and also during all the follow up period, i.e. a retrospective analysis of pacemaker data storage.

However, it was unfeasible to perform a reliable multivariate analysis because of the reduced number of patients, because of the lack of multiple risk factors, because of the contemporaneous usage in the same patients of acetate flecainide and of pacing prevention algorithms.

## Discussion and Conclusion

**Figure 3:**
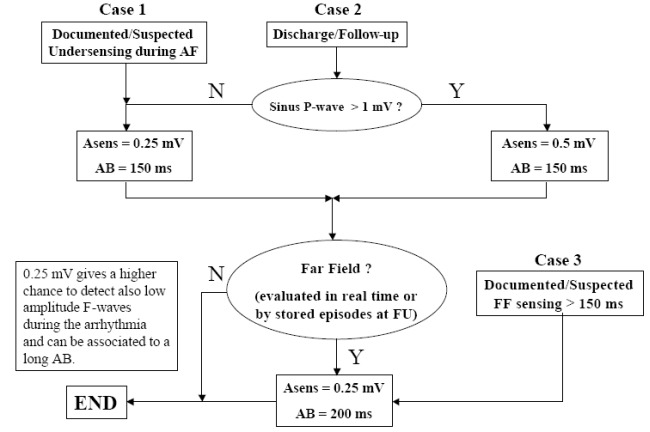
Decisional Flow-Chart

Presently, very few clinical randomised trials. [29-32] have compared the overlapping between pacemaker stored data and Holter monitoring ones, so that there is not a significant evidence of data about the reliability of these devices in properly recognising and monitoring supraventricular tachyarrhythmias. It’s also well known that bipolar leads often are not enough able to discriminate ventricular far field from normal P-wave amplitude or from wavelets of AF. An Atrial Blanking feature, long enough, may completely mask this phenomenon, but it also represents a blind interval for the atrial channel, sometimes subsequently resulting in a possible negative effect for an optimal arrhythmia detection. Recently, Nicotra [33] carried out a method for atria l sensing and blanking programming in order of guarantee reliable diagnostics in patients with paroxysmal AF. They proposed a decisional flow-chart based on the scanning of ventricular far field timing and amplitude that could focus and quantify this phenomenon for every kind of implanted device (Figure 3). This approach was also used in the present study during the follow-up.

Our data confirm the significant reliability of Selection^™^ 900E/AF2.0 Vitatron^®^ algorithms for detecting and monitoring AF. Moreover, we recorded a significant number of supraventricular tachyarrhythmia’s episodes, thus making stronger the statistical reliability of these data. Subsequently, we detected a progressive and significant reduction in AF burden and episodes. This decreasing in AF episodes and burden may be mainly related to the increase of atrial pacing, obtained by using a newly designed pacing overdrive algorithm responsible of atrial ectopic beats’ suppression in keeping with several trials [11-28].

Most of the discussed trials showed a lower reduction in AF episodes and burden if compared with our study (AF burden decreasing from 85% to 35% vs. a reduction from 93% to 0.3%; p<0.001; 95% confidence interval: -9.253 to -6.747). As showed in our recordings, the exceeding share of AF episodes and AF burden reduction is more likely to be entrusted to the newly designed four preventive pacing algorithms stored in the tested device (Selection^®^ 900E/AF2.0^®^ Vitatron^™^ pacemaker). Alternatively, as the data shown in Figure 2 seems to underline, i.e. that the efficacy of pacing for reducing AF episodes/day was evident after only six months of treatment, may suggest that also atrial remodelling could play a role. Electrical, mechanical and anatomical remodelling indicate structural alterations that, once established, may vanish any attempt to restore sinus rhythm. Atrial fibrosis is probably the most critical factor of the remodelling process and appears to be largely media ted by several mechanisms. Our clinical data indicate that these non pharmacological interventions may reduce, in a roundabout way, AF burden and episodes, probably interfering also with someone of those electrical and structural remodelling processes. It is possible, however, that having a very few patients, although followed for a median time of 24 months, we have overestimated the general possible reduction in AF episodes and burden because of a strong selection of our patients. In particular, we selected our patients on the basis of their atrial tachyarrhythmias’ onset mechanisms, and we tailored the available preventive pacing algorithms in each patient on the basis of each onset. This tailoring of pacing preventive algorithms on the basis of the different onsets in each patient, associated with the well known anti-remodelling effect of atrial overdrive pacing on atrial refractoriness dispersion, was the more important goal of our study and the likely reason of the observed marked reduction in AF episodes and burden. Therefore, these examined pacing algorithms may represent an effective therapeutical options to contrast the nearly inevitable progression of this arrhythmia towards its permanent form.

The AF antiarrhythmic drugs therapy represents the first and more effective therapy, although it is well known that it is not always effective and free from side effects. In our patients, implanted for a sick sinus syndrome and at least partially resistant to AF pharmacological treatment, new interventional non-pharmacological solutions, such as tailored antiarrhythmic pacing algorithms, may represent a further effective therapeutical option. Recent analysis of AFFIRM [33] and RACE trial [34] showed that, in patients older than 65 years, with well defined risk factors, a therapeutical strategy based on ventricular rate control is not inferior to a strategy of maintaining sinus rhythm (rhythm control). However, this issue is still debated. Indeed, other clinical trials and sub-analysis [35-40] showed that sinus rhythm restoration has to be preferred compared to rhythm control.

In conclusion, our experience suggests the reliability of well tailored pacing algorithms on AF control in selected patients with brady-tachy syndrome, refractory to pharmacological rhythm control. However, we should say that this study is partially limited by the small and highly selected sample used, that reduces the possibility of translating theses results to general population. Moreover, this was not a truly randomized, controlled study, and so interpretation of the clinical significance of the reduction of AF episodes, as also shown however by several other trials in International Literature, is uncertain, thus not allowing us for making really definitive conclusions. The main information, however, we can derive from this trial is that we should better evaluate, before every pacemaker implantation, the different AF onsets of each patient eligible for such a procedure, because only a really profound knowledge of the different onset mechanisms of atrial tachyarrhythmias in each subject may give us the possibility of choosing a really effective implantable device for these sometimes very different patients, thus obtaining the maximum effective clinical results from these pacing algorithms in terms of reduction of AF episodes and burden.

## Figures and Tables

**Table 1:**
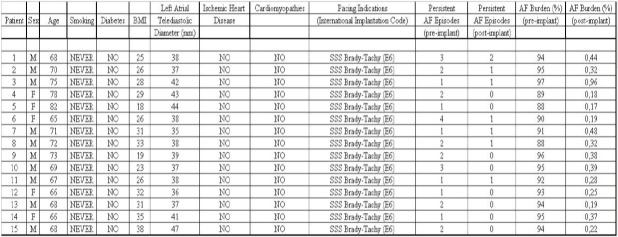
Patients baseline caracteristics and follow-up
